# Editorial: Dynamic Biomarkers of Response to Anti-Immune Checkpoint Inhibitors in Cancer

**DOI:** 10.3389/fimmu.2021.781872

**Published:** 2021-10-21

**Authors:** Said Dermime, Maysaloun Merhi, Taha Merghoub

**Affiliations:** ^1^ National Center for Cancer Care and Research, Hamad Medical Corporation, Doha, Qatar; ^2^ Translational Cancer Research Facility, Translational Research Institute, Hamad Medical Corporation, Doha, Qatar; ^3^ College of Health and Life Sciences, Hamad Bin Khalifa University, Doha, Qatar; ^4^ Ludwig Collaborative, Swim Across America Laboratory, Memorial Sloan Kettering Cancer Center (MSK), New York, NY, United States; ^5^ Parker Institute for Cancer Immunotherapy, Memorial Sloan Kettering Cancer Center (MSK), New York, NY, United States; ^6^ Human Oncology and Pathogenesis Program, Department of Medicine, Memorial Sloan Kettering Cancer Center (MSK), New York, NY, United States; ^7^ Department of Medicine, Weill Cornell Medicine, New York, NY, United States

**Keywords:** immune checkpoint inhibitors (ICI), programmed cell death protein 1 (PD1), programmed cell death ligand 1 (PD-L1), dynamic biomarkers, irAEs

Immune checkpoint blockade (ICB) has been approved as first- or second-line therapy options in a broadening range of metastatic cancer and is increasingly explored in the treatment of early stage tumors. However, clinical responses are limited to a small group of patients and potentially long-lasting responses were observed in 10% to 40% of cancer patients, depending on the malignancy subtype (1, 2). Considerable efforts have been made to identify predictive factors of response to ICB with the aim to use this therapy in patients with a high probability of response and to avoid exposing non-responder subjects to their potential side effects. Whilst a range of biomarkers have been investigated, their predictive potential remains unsatisfactory. In current clinical practice only PD-L1 expression in tumor tissues is used as predictive biomarker of response to PD-1 immune checkpoint blockade. However, a small proportion of patients with absent PD-L1 tumor expression may still respond to PD-1 blockade, making it difficult to restrict prescription of these therapies solely based on this biomarker. Hence, the search for novel biomarkers of response to checkpoint inhibitors remains an unmet need. One promising emerging approach is to focus on dynamic biomarkers, which would allow, when tested in the patient early after exposition to the therapeutic agents, to identify those patients presenting an immune response failure. The study of the dynamics of the immune system and of the tumor under immune checkpoint blockade shed light on their mechanisms of activity. Indeed, some immune pathways induced by ICB therapy may affect anti-tumor activity, whilst others may correlate with immune related adverse events (irAE) rather than with response. Moreover, tumor-intrinsic mechanisms of immune escape may develop following ICB and will consequently affect treatment outcome. We have recently summarized the dynamics of the immune system and of the tumor under immune checkpoint blockade. We emphasized the importance of studying mechanisms influencing response to ICB and focused on the multitude of immune cells subsets (including effector and immunosuppressive T cells and B cells subsets) that were shown to be impacted by CTLA-4 and PD-1/PD-L1 blockade monotherapy (3). In this Research Topic, we compiled reviews and original research articles reflecting the current advances in the study of dynamic biomarkers as predictors of the response to checkpoint inhibitors therapies in cancer. Four complimentary areas are addressed in this topic.

## Monitoring Specific Anti-Tumor Immune Responses to Tumor Associated Antigens and/or Neoantigens Before and During Immune Checkpoint Inhibitors Treatment

It has been demonstrated that Tumor Mutational Burden (TMB) is a useful biomarker to predict the response to immunotherapy in cancer patients. Usuda et al. indicated that high TMB and high PD-L1 expression can predict a favorable outcome of ICB therapy in lung cancer patients. Nie et al. reported that an increased TMB profile in elderly melanoma patients may restore the age-related immune dysfunction leading to favorable immune response to anti-PD-1/anti-PD-L1 therapy comparable between patients younger or older than 75 years old. An integrative analysis by Mo et al. showed that the gene mutation CTNNB1 (catenin beta-1), which is associated with a better prognosis in multiple tumors, would help in improving the clinical outcome of immunotherapy in patients with hepatocellular carcinoma (HCC). Interestingly, this study has reported a significant increase in tumor infiltrated NK cells in the HCC mutation group compared to wild-type group. Moreover, CTNNB1 mutation was associated with a downregulation of 4 immunoinhibitory genes including the NK cells immune checkpoint receptor CD96. Chen et al. showed that cyclin D1 (CCND1) amplification triggered multiple immunosuppressive hallmarks and may be useful to predict immune response to ICB. Bioinformatic and biostatistical analysis tools have been shown useful to predict gene signatures associated with clinical benefit of immune checkpoint blockade therapy. Indeed, the group of Chen R. L. have established an immune-related risk signature in squamous-cell lung cancer (SQLC) by identifying 8 immune-related genes that correlated with immune cells infiltration and clinical outcome in SQLC patients. Moreover, Chen S. et al. have developed an immunotherapy-responsive model including 10 prognostic genes that might be helpful to predict response to ICB in patients with metastatic urothelial carcinoma. Xue et al. have established and validated a signature profile including 23 immune related gene pairs (IRGP) associated with higher overall survival in cutaneous melanoma (CM) patients. This study showed that low 23-IRGP value with increased expression of PD-1/PD-L1 were associated with a better prognosis and suggested that the 23-IRGP could be a useful predictive biomarker of response to ICB therapy in CM patients. These findings indicate that immune related genes can be explored as promising biomarkers for predicting immunotherapy clinical outcomes. However, Wu S. et al. reported that mismatch repair deficiency and microsatellite instability are not sensitive predictive biomarkers in triple negative breast cancer and that the investigation of concomitant expression of immune checkpoints such as PD-L1 and LAG-3 in tumor tissue will be more helpful for establishing a successful ICB treatment *via* dual blockade antibodies therapy. Wu D. et al. identified highly immunogenic clonal neoantigen (in an epidermal growth factor receptor (EGFR)-mutated NSCLC patient benefitting from anti-PD-1 blockade after acquiring resistance to EGFR-Tyrosine kinase inhibitor (TKI) therapy. This study validated 4 clonal neoantigens, including 2 derived from EGFR 19del, 1 from Tumor protein P53 A161T (TP53 A161T) and 1 from DENN domain containing D6B R398Q (DENND6B R398Q) and emphasized the urgent need of robust approaches to explore specific clonal neoantigens derived from driver mutations to predict clinical benefit of ICB in EGFR-TKI resistant NSCLC patients. Zhang et al. showed a 20% decrease of 4 serum tumor biomarkers; carcinoembryonic antigen (CEA), cancer antigen 125 (CA125), cytokeratin 19 fragment (CYFRA21-1) and squamous cell carcinoma related antigen (SCC-Ag) at 6 weeks after anti-PD-1/PD-L1 therapy compared to baseline and this was associated with an improved prognosis of late-stage NSCLC patients. The group of Yao Chen stressed the need of predictive biomarkers to properly select those patients resistant to standard therapies but might benefit from a novel combination involving ICB. This group showed that high circulating-free DNA (cfDNA), high circulating tumor DNA (ct-DNA) and specific mutations (such as MIKI67 and hyper-progressive disease-related gene mutations) are associated with poor outcome in late-stage NSCLC patient treated with combination of ICB and anti-angiogenic therapy. The NY-ESO-1 cancer testis antigen has been shown to be expressed in several tumors. It is a highly immunogenic tumor-specific antigen inducing both humoral and T cell responses and it is considered as an attractive target for immunotherapy. In fact, NY-ESO-1 can be considered as a dynamic biomarker and a potential target of immunotherapy (4). Indeed, it has been demonstrated that melanoma patients treated with ipilimumab had an increased rate of NY-ESO-1-specific immunity that was associated with improved clinical benefit of the treatment, especially in patients developing both NY-ESO-1-specific antibody and specific CD8+ T cells (5). We have shown that immunological monitoring of NY-ESO-1-specific T cells response is a useful biomarker of response to anti-PD-1 treatment. We have characterized the expression of immunological markers before and after anti-PD-1 treatment in a patient with recurrent Head and neck Squamous cell carcinoma (HNSCC) who showed a transient regression and stability of the tumor after ICB for over 7 months. We observed that clinical responsiveness to anti-PD-1 treatment correlated with immunity to NY-ESO-1. Our results showed that NY-ESO-1-specific T cells response was increased after the fifth cycles of treatment (stable disease) but had a significant decline at progression, and that PD-1+ T cells population was markedly reduced after anti-PD-1 treatment (6). Recently, we have characterized a dynamic NY-ESO-1- specific T cells population that was significantly increased at treatment response in a patient with a metastatic gastric cancer who displayed a long-lasting response to combined radio-immunotherapy (4).

## Identification of Phenotypic Markers In Immune Cells Before and During Immune Checkpoint Inhibitors Treatment

Several studies have demonstrated that phenotypic characteristics of immune cells play an important role in predicting the prognosis of cancer patients and their response to different anti-cancer therapies, specifically ICB. Indeed, in a patient with metastatic gastric cancer who responded to combined radio-immunotherapy, we have identified a peripheral CD107+ cytotoxic T cells subset within a specific CD8/HLA-A2-NY-ESO-1 T cell population that was low at disease progression (2.5%), markedly increased at disease resolution (12.9%) and significantly decreased again at disease re-progression (3.6%) (4). In a retrospective study including 79 cancer patients who received anti-PD-1 therapy, Yuan et al. observed that responders had lower number of peripheral B cells compared to patients who developed post-treatment progressive disease. These findings suggest that blood B cells might be a potential biomarker for anti-PD-1 therapeutic outcome. Interestingly, Araujo B. de Lima et al. suggested that patients responding to ICB present a pre-existing immune cells signature qualified as “favorable immune periphery” useful to predict individuals who will benefit from ICB therapy. This group characterized peripheral immune cells phenotypic profile at baseline, 6 then 20 weeks after starting ICB in 33 patients with solid tumors. The results showed that high levels of circulating CD8+ PD-1+ T cells, of effector-memory CD8+ T cells, abundance of dendritic cells (DCs) and low levels of myeloid-derived suppressor cells and monocytes at baseline correlated with good prognosis and response to ICB treatment. Zielinski et al. discussed novel technologies that have the potential to accelerate the characterization of functional phenotype of immune cells before and during anti-cancer therapies to evaluate patients’ response and to predict their prognosis. This study suggests high throughput approaches including multi-omics, single cells sequencing, flow cytometry, mass cytometry and bioinformatics for the exploration of immune cells dynamics and discovery of novel cancer biomarkers. Kidman et al. also pointed out the application of sequencing and single-cells analysis in characterizing dynamic changes in the T cell receptor (TCR) repertoire during ICB therapy to evaluate treatment outcome and stratify patients according to predicted beneficial response. Briefly, this study reported that anti-CTLA-4 treatment increases TCR diversity and clonal expansion of the peripheral and tumor-infiltrating lymphocytes. Moreover, this study suggested that the expression of PD-1+ blood T cells and the expansion of intra-tumoral TCR clonotypes could be predictive biomarkers of response to PD1/PD-L1 blockade. The same group has identified in murine model a subset of antigen specific CD8+ T cells correlating with a successful response to ICB (Principe et al.). Hence, this study suggested that increased effector memory antigen-specific CD8+ cytotoxic T cells in the presence of reduced regulatory T cells (Tregs) within tumors is predictive of response to anti-PD-L1 and anti-CTLA-4 treatment. Hübbe et al. discussed strategies described to stimulate endogenous DCs for improved anti-tumor immune response to ICB. Accordingly, the presence of adequately activated DCs could be considered as a biomarker for response to ICB since engagement of stimulated DCs will have an impact on the expansion, proliferation and priming of antigen-specific anti-tumor T cells. Finally, the expression levels of the inhibitory proteins PD-1 and CTLA-4 by T cells can be a useful prognostic biomarker associated with cancer immunity. Indeed, based on pan-cancer analysis of large cancer datasets, Liu et al. showed that a differential expression profiles of these immune cells inhibitory receptors are observed by cancer type and are associated with several parameters that play a key role in response to immunotherapy including tumor cell infiltration and TMB.

## Identification of Cytokines/Chemokines Specifically Generated Before and During Immune Checkpoint Inhibitors Treatment

Cytokines and chemokines are biomarkers that play a pivotal role in immune cells activity. It has been shown that differential expression levels of these biomarkers were observed during ICB and can be helpful in monitoring the clinical outcome. We have shown that several cytokines/chemokines involved in immune activation were upregulated after ICB (nivolumab) treatment of HNSCC patient; 2 biomarkers were reduced at progression [interleukin (IL)-10: ****p < 0.0001 and CX3CL1: ****p < 0.0001] (6). On the other hand, some cytokines/chemokines contributing to immune inhibition were downregulated after nivolumab treatment; 2 biomarkers were increased at progression (IL-6: ****p < 0.0001 and IL-8: ****p < 0.0001) (6). We have also shown that downregulation of IFN-γ, tumor necrosis factor-α (TNF-α) and few interleukins (IL-2, IL-5 and IL-6) concomitant with an upregulation of perforin, soluble FAS, macrophage inflammatory protein-3α (MIP-3α) and C-X-C motif chemokine 11/Interferon–inducible T Cell Alpha Chemoattractant) correlated with disease resolution in a metastatic gastric patient treated with combined radioimmunotherapy (4).

## Biomarkers Associated With Treatment Related Toxicity After Treatment With Immune Checkpoint Inhibitors

As a consequence of their activity as modulators of the immune response, ICB can exert immune-related adverse events (irAEs) potentially leading to severe outcome and discontinuation of therapy. In addition to most common side effects including dermatological (reticular, erythematous rash), gastrointestinal (diarrhea, colitis), and endocrine (thyroid, adrenal, pituitary glands) effect, rarely described hematological irAEs associated with ICB therapy (thrombocytopenia, aplastic anemia, etc.) are emphasized in a systematic review of 49 case report articles which also summarized the current approaches used to manage this toxicity (Omar et al.). The pattern and the onset of irAEs differ by ICB treatment and by cancer type resulting in a highly variable clinical presentation considered as a complicated challenge for oncologists. Therefore, the identification of biomarkers for early recognition and appropriate management of ICB-iRAEs is critical for clinical practice. Indeed, several studies have urged the need of accurate irAEs biomarkers and highlighted the difficulty to distinguish them from regular toxicities. Pringle et al. reported an infiltration of CD4+ and CD8+ T cells and an increased expression of proliferative (ki67+) and senescent (p16+) epithelial cells in the salivary gland tissue of a NSCLC patient who was suffering from post-durvalumab (anti-PD-L1) hyposalivation. The review article by Hommes et al. presents a combination of biomarkers that can be useful to predict ICB toxicity, including blood-based (neutrophil-to-lymphocyte ratio, absolute lymphocyte count, lymphocytes, autoantibodies and various serum cytokines/chemokines), immunogenetic (single nucleotide polymorphisms, human leucocyte antigen subtypes) and microbial biomarkers (microbiome composition). However, this study highlights the limitations related to the specificity and accuracy of these biomarkers. Rose L. M. et al. evaluated the association between tumor type, pre-treatment clinical parameters and the occurrence of irAEs in cancer patients treated with ICB as a monotherapy or in combination. This retrospective study suggests that demographic parameters (female, obesity) and preexisting clinical factors, such as high eosinophils or white blood cells counts and pre-existing autoimmune disease, could help identify cancer patients at higher risk of developing irAEs (Rose L. M. et al.). An interesting review by Xu et al.). summarized the key potential biomarkers from the tumor microenvironment, peripheral blood, targeted organs, host-related demographic parameters and treatment history, associated with an increased incidence of ICB-related toxicities. However, with the modification of the tumor status, tumor microenvironment, immune response and potential concomitant therapies, dynamic monitoring would be required for several biomarkers to manage the toxic outcome accurately and adequately. In conclusion, the identification of specific biomarkers for ICB-related toxicities is still at exploratory research stage and needs further and larger investigations. In an attempt to overcome the side effects of ICIs monoclonal antibodies, researchers are trying to target checkpoint inhibitors such as PD-L1 using novel immunotherapeutic approaches. In a phase I clinical trial, Jørgensen et al. showed that subcutaneous vaccination with IO103 peptide against PD-L1 induced significantly high immunogenicity with low-grade and reversible toxicity in multiple myeloma patients.

## Conclusion

The study of the dynamics of the immune system and tumor microenvironment during ICB is crucial to predict favorable response and to manage undesirable adverse events. Dynamic predictive biomarkers of ICB identified so far are still at exploration phase and have limited reliability on a patient basis. The development of models combining multiple variables dynamics and using novel system approaches such as multi-omics, single cells analysis and rigorous biostatistics and bioinformatics tools is key in the identification of reliable predictive dynamic biomarkers to pave the way towards a more personalized, beneficial and safer ICB therapy.

## Author Contributions

SD, TM, and MM developed and designed the topic, selected authors, and reviewers, reviewed submitted articles and supported their publication. MM, TM, and SD wrote the editorial. All authors revised the manuscript and approved the submitted version.

## Funding

SD and MM are supported by the Medical Research Center at Hamad Medical Corporation under the approved grant MRC-01-19-300. TM is supported by the Ludwig Institute for Cancer Research, NIH/NCI Cancer Center Support Grant P30 CA008748, the Parker Institute for Cancer Immunotherapy and Swim Across America.

## Conflict of Interest

TM has acted as a consultant for Immunogenesis, Immunos Therapeutics and Pfizer, has received research support from Adaptive Biotechnologies, Aprea, Bristol Myers Squibb, Infinity Pharmaceuticals, Kyn Therapeutics, Leap Therapeutics, Peregrine Pharmaceuticals and Surface Oncology, is listed as a co-inventor on patents relating to the use of oncolytic viral therapy, alphavirus-based vaccines, antibodies targeting CD40, GITR, OX40, PD-1 and CTLA-4 and neo-antigen modelling, and is a cofounder of and holds an equity in IMVAQ Therapeutics.

The remaining authors declare that the research was conducted in the absence of any commercial or financial relationships that could be construed as a potential conflict of interest.

## Publisher’s Note

All claims expressed in this article are solely those of the authors and do not necessarily represent those of their affiliated organizations, or those of the publisher, the editors and the reviewers. Any product that may be evaluated in this article, or claim that may be made by its manufacturer, is not guaranteed or endorsed by the publisher.
